# Elucidation of the Origin of the Monumental Olive Tree of Vouves in Crete, Greece

**DOI:** 10.3390/plants10112374

**Published:** 2021-11-04

**Authors:** Aureliano Bombarely, Andreas G. Doulis, Katerina K. Lambrou, Christos Zioutis, Evi Margaritis, Georgios Koubouris

**Affiliations:** 1 Department of Bioscience, Universita degli Studi di Milano, 20133 Milan, Italy; abombarely@ibmcp.upv.es; 2 Instituto de Biologıa Molecular y Celular de Plantas (IBMCP), UPV-CSIC, 46022 Valencia, Spain; 3 Hellenic Agricultural Organization (ELGO) DIMITRA, Institute of Olive Tree, Subtropical Crops and Viticulture, 73134 Chania, Greece; doulis@elgo.iosv.gr (A.G.D.); lamprou@elgo.iosv.gr (K.K.L.); zioutisch@gmail.com (C.Z.); 4 Science and Technology in Archaeology and Culture Research Center (STARC), The Cyprus Institute, Nicosia 2121, Cyprus; e.margaritis@cyi.ac.cy

**Keywords:** *Olea europaea*, whole genome DNA resequencing, Illumina, RNA-Seq, gene space comparison

## Abstract

The olive tree of Vouves in Crete, is considered the oldest producing olive tree in the world with an estimated age exceeding 4000 years. In the present study, we sequenced two samples (from the bottom and the top of the tree) to elucidate the genetic relation of this ancient tree with other olive cvs as well as to gain some insights about its origin. Our results showed that both samples have different genetic origins, proving that this ancient tree has been grafted at least one time. On the basis of whole genome sequences the sample from the top of the Vouves tree showed relation of the same order than half-siblings to one accession corresponding to the present-day Greek cv ‘Mastoidis’. Nevertheless, in the framework of a microsatellite analysis it was found to cluster with the ‘Mastoidis’ samples. The Vouves rootstock (bottom sample) showed a clear grouping with the oleaster samples in a similar way to that of ‘Megaritiki’ Greek cv although it does not show any signal of introgression from them. The genomic analyses did not show a strong relation of this sample with the present-day Greek cvs analyzed in this study so it cannot be proved that it has been used as a source for cultivated olive tree populations represented by available genome sequences. Nevertheless, on the basis of microsatellite analyses, the Vouves rootstock showed affinity with two present-day Greek cvs, one “ancient” rootstock from continental Greece as well as monumental trees from Cyprus. The analysis of the impact of the variants in the gene space revealed an enrichment of genes associated to pathways related with carbohydrate and amino acid metabolism. This is in agreement with what has been found before in the sweep regions related with the process of domestication. The absence of oleaster gene flow, its old age and its variant profile, similar to other cultivated populations, makes it an excellent reference point for domestication studies.

## 1. Introduction

Olive (*Olea europaea* subsp. *europaea*) is the dominant tree crop in the Mediterranean countries. In fact, over the 90% of the global olive production is realized in this region [1]. The products of this emblematic crop, namely olive oil and table olives are popular in the framework of a healthy lifestyle [2,3]. Even in parts of the world far from its traditional cultivation area, such as Eastern Asia, Australia and America, there is a strong interest in growing olive trees and consuming its nutritional and rich in biological value products. The world annual gross production value exceeds 18.3 billion dollars for 2014, 2015 and 2016 [4] depicting the significant contribution in the economic life of producer countries. Even though that the olive tree has gained a lot of attention for the health benefits of its products as well as for its central role in history and culture, the roots of its domestication have not been unambiguously identified [5]. Even in our days, there is an open debate about whether one major domestication event was realized in the Eastern Mediterranean followed by subsequent dispersion westwards [6] or more than one independent domestication incidents shaped the richness of olive genetic resources available to date [7]. In any case, it is common belief that the wild olive *Olea europaea* var. *sylvestris* is the ancestor of the cultivated olive *Olea europaea* var. *europaea* [8,9,10]. Interestingly, wild olive trees still survive in some Mediterranean forests [11].

Archaeological studies in Greece have identified olive pollen dating to 7000 BC [12] while they suggest that human was firstly attracted by the olive wood during the third millennium B.C. and that fruit use followed the transformation of olive bush to a tree through pruning [13]. Indeed, early historical references on the use of fruit for its oil content appear much later [14].

The olive tree was considered as sacred in ancient Greece and it was the highest prize for the winner athletes in the Olympic Games [15]. In an attempt to revive and honor this tradition, during the modern Olympic Games held in Greece in 2004, the winners were crowned with an olive wreath from the millennial olive tree of Vouves in Crete, which is considered the oldest producing olive tree in the world (https://en.wikipedia.org/wiki/Olive_tree_of_Vouves, accessed on 1 August 2021) with an estimated age exceeding 4000 years. The oldest indication for the incidence of olive oil based on analysis of residue on pottery is dated at the 4th millennium in the Gerani Cave in Crete [16]. In the case of Prepalatial Chrysokamino, it was found that olive oil was used to cover wine for preventing its oxidation to vinegar [17]. In another instance, olive oil was noticed in the Early Minoan I site of Aphrodite’s Kephali [18]. After a long period of time, Late Bronze Age findings were reported from the cemetery of Armenoi [19] and Pseira [20] in Crete. Taking into consideration all these findings it is concluded that proof for the production and use of olive oil in Crete surely precedes the Late Bronze Age.

The millennial olive tree of Vouves in Crete is considered to consist of a Greek cv resembling ‘Mastoidis’ grafted onto an unknown ancient rootstock yielding a present-day “monumental” tree. A three-dimensional model of its trunk has been elaborated while the overall physiognomy is discussed [21]. Nevertheless, the tree trunk is presented as a compact object without differentiating between the two parts (top vs. bottom rootstock). In ancient times, olive domestication was based on selection of wild trees with desirable properties and grafting on other trees, as reported by Theophrastus more than 2000 years ago [22].

The study of ancient olive trees is valuable for detecting unknown genetic resources and for shedding light in the historical processes of olive domestication [23]. Previous studies on genotyping of old olive trees revealed ancient cvs and confirmed the selection of cvs [24] as well as rootstocks [25].

The similarity in genetic structure of naturally growing populations with the suckers of old cultivated trees implies that wild trees were used as rootstocks [26]. Pollen archaeological studies suggest that a cultivation process seems to have occurred in the Aegean (Crete)—whether as an independent large-scale management event or as a result of knowledge and/or seedling transfer from the southern Levant around the fourth millennium BC [27].

In this case, 35 species have been described in the Olea genus in which the most popular one is *O.* *europaea*. This species has been divided into six subspecies: *Europaea*, cultivated in the Mediterranean basin; *Laperrinei*, native to Saharan massifs; *Cuspidata*, widely distributed from South Africa to Southern Egypt, and from the Arabian Peninsula to Southwest China; *Guanchica*, endemic to the Canary Islands; *Maroccana* from Morocco and *Cerasiformis* native to Madeira [28]. The cultivated olive tree (*O. europaea* subsp. *europaea*) has a diploid genome with 46 chromosomes (2n = 2x = 46) and a variable genome size ranging from 1.65 [28] to 2.21 Gb [29]. At the time of this publication in 2021, five de-novo olive tree genome assemblies from four different varieties are available: *O. europaea* subps. *europaea*, cv. “Farga” version Oe6 [30] and its improvement, Oe9 [31]; *O.* *europaea* var. *sylvestris* version Oe451 [32]; *O. europaea* subps. *europaea*, cv. “Picual” version Oleur0.6.1 [33] and the most recent assembly, *O. europaea* subps. *europaea*, cv. “Arberquina” version Oe_Rao [34]. Also, some genotyping-by-sequencing studies are available such as [35].

**Figure 1:**
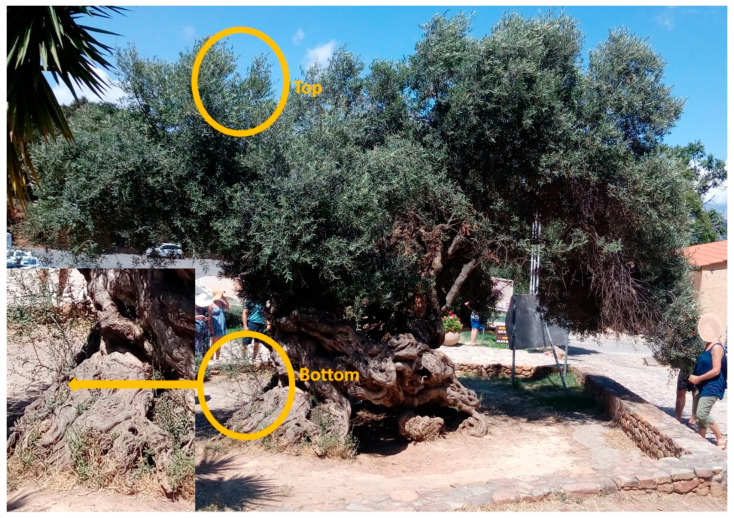
Vouves monumental olive tree picture. The places from which the samples were taken are marked in yellow.

In the present study, we sequenced the genome of the Olive Tree of Vouves, which is considered the oldest producing olive tree in the world. The upper part of the tree, the scion, producing fruit and the lower part of the tree, the rootstock, providing the roots for supplying water and nutrients, were sequenced separately (Figure 1). Further, in order to gain an initial understanding into the relative placement of the two Vouves parts within the overall present-day Greek cv diversity landscape, a separate microsatellite (SSR) analysis was conducted. For that, samples were selected so as to span placement across the SSR similarity dendrogram from [36] and analyzed anew. In total 17 samples were genotyped including 12 present-day Greek cvs (alphabetically; Adramytini, Amfissis, Chalkidikis, Gaidourelia, Karydolia, Koroneiki, Mastoidis, Megareitiki, Pierias, Pikrolia, Tragolia and Vasilikada), the two Vouves samples, one “ancient” rootstock from Peloponnese and one *Olea europaea* subsp. *cuspidata* (Wall. and G. Don) Cif. sample as an outgroup).

Our aims were to (i) verify that the tree is consisted of two different genotypes united through grafting, (ii) reveal the genetic identity of the two genotypes forming this historical and millennial tree, (iii) elucidate their genetic relation with other olive cvs, especially of Greek origin, (iv) characterize their genetic differences in relation to the *Olea europaea* var. *sylvestris* reference genome and (v) propose sound hypotheses on the origin of the Vouves monumental olive tree.

## 2. Results and Discussion

### 2.1. Resequencing, Mapping and Variant Calling with the Olea europaea var. sylvestris Reference Genome

Leaves from two different parts of the tree (bottom and top) were sampled for the DNA extraction, library preparation and short read whole genome sequencing. Subsequently, 63.04 and 75.97 Gb of pair end reads were obtained from the bottom and top Illumina libraries. Then, 411.04 and 496.72 million reads accounting for 58.01 and 70.87 Gb, respectively, were mapped to the reference genome (*Olea europaea* var. *sylvestris* version Oe451). Indeed, 95.49% and 96.71% of the reference genome were covered by at least one read from the bottom and top samples, leaving 51.48 and 37.61 Mb of the reference uncovered by any read for each of the samples, respectively. The average mapping coverage was 51.39 and 62.65 X, respectively. The variant calling of the two different samples delivered 23.26 (2.09 variants/100 bp) and 19.54 (1.76 variants/100 bp) millions of variants for bottom and top samples, respectively. The comparison of both accessions with the reference delivered 7.24 and 4.61 million of homozygous variants of which both samples shared 1.66 million of variants. Both samples were compared with the Greek samples resequenced in [33] and reanalyzed in this work. These samples included ‘Kalamon’, ‘Koroneiki’, ‘Mastoidis’, ‘Mavreya’, ‘Megaritiki’ and ‘Myrtolia’. Results are summarized in Table 1.

The levels of heterozygosity were similar between all the samples, ranging from 1.31 heterozygous variants/100 bp of bottom of the Vouves tree to 2.16 of heterozygous variants/100 bp ‘Megaritiki’. The high levels of heterozygosity are concordant with other projects in which an olive genome was sequenced such as the ‘Farga’ (5.4%) [30] and ‘Picual’ genomes (2.02%) [33] whereupon similar values were determined. In a different work, genotyping of an olive panel using Genotyping-By-Sequencing (GBS) delivered values ranging from 1.28% of a cv called ‘Zhonglan’ to 6.36% of the Italian cv ‘Nociara’ [35], including some Greek samples such as ‘Koroneiki’ with 2.19%. Olive genome heterozygosity is much higher than in other tree crops. For example, the apple (*Malus domestica*) variety ‘Golden Delicious’ is considered highly heterozygous reaching values of 0.32 heterozygous variants each 100 bp [37]. Peach (*Prunus persica*) is another example of a tree crop where the average heterozygosity for cvs and wild relatives are 0.07% and 0.25%, respectively [38]. Avocado trees (*Persea americana*) have heterozygosity levels of the same order with olive trees. Indeed, estimated heterozygosity of the ‘Hass’ variety is 1.05% [39].

### 2.2. Origin of the Vouves Monumental Olive Tree in the Context of Olive Domestication

**Figure 2:**
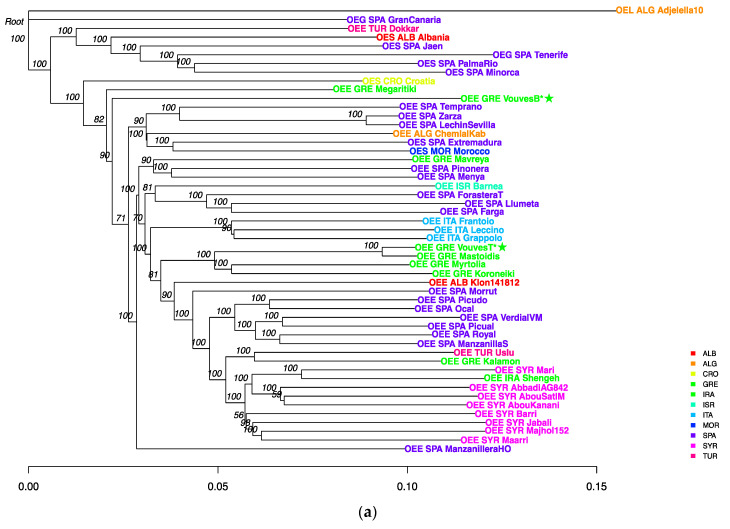

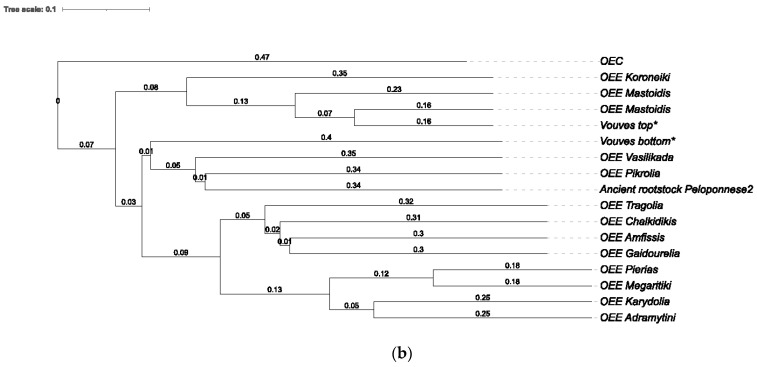
(**a**) Phylogenomic NJ tree made with the 71,040 filtered biallelic SNPs produced with whole genome resequencing data. Taxa names encode the subspecies (OEL, for *Olea europaea* subsp. *laperrinei*; OEG, *Olea europaea* subsp. *guanchica*; OES, *Olea europaea* var. *sylvestris* and OEE, *Olea europaea* subsp. *europaea*), country of origin, also with different colors [ALB (red), Albania; ALG (orange), Algeria; CRO (yellow), Croatia; GRE (light green), Greece; IRA (green), Iran; ISR (green-blue), Israel; ITA (light blue), Italy; MOR (blue), Morocco; SPA (purple), Spain; SYR (light purple), Syria and TUR (pink), Turkey] and variety name (*n* = 51). Bootstrap values are in cursive over their respective nodes. Target samples (Vouves Bottom and Top) have been marked with an asterisk (**b**) UPGMA similarity dendrogram of 12 present-day Greek cvs, the two Vouves samples and one “ancient” olive-tree genotype based on Jaccard’s index (*n* = 16). Distances based on Jaccard’s index are indicated on each branch of the tree. *Olea europaea* subsp. *cuspidata* (Wall. and G. Don) Cif. (denoted as OEC) was employed as an outgroup. Other taxa names correspond to the *Olea europaea* subsp. *europaea* (OEE) cvs (data from [36] reprocessed within the framework of present study). Distances are indicated above each tree branch.

All RNASeq data from NCBI SRA project PRJNA525000 [6] as well as the Whole Genome DNA Resequencing (WGR) data from the SRA project PRJNA556567 [33] were used in an effort to propose sound hypotheses regarding the origins and phylogenomic/phylogenetic relations of the Vouves’ olive tree. The first dataset contains 56 samples of wild and cultivated olive trees from 14 different countries across the Mediterranean basin. The second dataset, eventually used to produce Figure 2a, contains 41 different cultivated varieties (*Olea europaea* subsp. *europaea*) as well as 10 wild accessions (i.e., a total of 51 taxons), including different subspecies such as *laperrinei* and *guanchica* and wild *Olea europaea* subsp. *europaea* varieties (*Olea europaea* subsp. *europaea* var. *sylvestris*), syn *Olea europaea* var. *sylvestris* (also called oleasters). After the read mapping, variant calling and filtering, 299, 435 biallelic SNPs were obtained for 117 individuals. Subsequently, samples coming from RNASeq and WGR were compared so as to assess if it is feasible to combine data sets produced from two different methodologies (i.e., RNASeq and WGR). It was found that samples clustered by methodology and not by origin or cv (Figure S1). Consequently, and based on this result, data derived from RNASeq analyses were filtered out, retaining only the WGR data for subsequent analyses. An additional filtering was applied to remove linked variants obtaining a total of 71,040 biallelic SNPs.

The distance tree produced using these variants was employed to construct the phylogenomic NJ tree depicted in Figure 2a. Accession (*Olea europaea* subsp. *laperrinei*) termed ‘Adjelella10’ was employed as an outgroup. In Figure 2a it can be observed that accession ‘Gran Canaria’ is sister to the outgroup accession as is expected for a different subspecies (*Olea europaea* subsp. *guanchica*) although the other *guanchica* accession, ‘Tenerife’, is nested with the oleaster accessions (*Olea europaea* var. *sylvestris*). Accession ‘Dokkar’ is also nested with the oleaster accessions. The rest of the accessions are part of the same clade. There are three oleaster accessions nested with the cultivated accessions, ‘Croatia’ acting as an outgroup of the cultivated accessions and ‘Extremadura’ and ‘Morocco’ that are nested with three accessions which originate from southern Spain (‘Temprano’, ‘Zarza’ and ‘Lechin de Sevilla’) and the Algerian accession (‘Chemlal De Kabylie’).

In the phylogenomic tree it can be seen that the Vouves tree bottom sample (more than 4000-year-old) is external to all the cultivated samples except for ‘Megaritiki’. The Vouves top tree sample is sister to the ‘Mastoidis’ accession and it clusters with other present Greek samples. The Italian samples (‘Frantoio’, ‘Leccino’ and ‘Grappolo’) are a monophyletic group as well as all the Syrian and Iranian accessions. The Spanish samples are divided into four groups. The first one is nested with two oleaster accessions (‘Extremadura’ and ‘Morocco’). The 1 (‘Pinonera’ and ‘Menya’) is sister to the Greek accession ‘Mavreya’. The third one (‘Farga’, ‘Llumeta’ and ‘Forastera de Tortosa’) are sister to the Israel accession ‘Barnea’. The fourth group contains accessions from southern Spain and is sister to the Syrian/Iranian clade. The ‘Kalamon’ accession is sister to the Turkish accession ‘Uslu’. In the UPGMA similarity tree produced with 11 SSR loci (Figure 2b) it can be seen that ‘Vouves bottom’ exhibits high similarity with another “ancient” rootstock from the Greek province of Peloponnese. This province is isolated from Crete by sea. Further, the Peloponnese rootstock shares high similarity with present-day Greek cvs “Pikrolia” and “Vasilikada’. This is in full agreement with a subsequent—yet unpublished—populational study involving few hundred olive tree samples from all over Greece genotyped with SSR markers. In this study each Greek cv is represented by a series of newly analyzed independent genotypes (data not shown).

**Figure 3:**
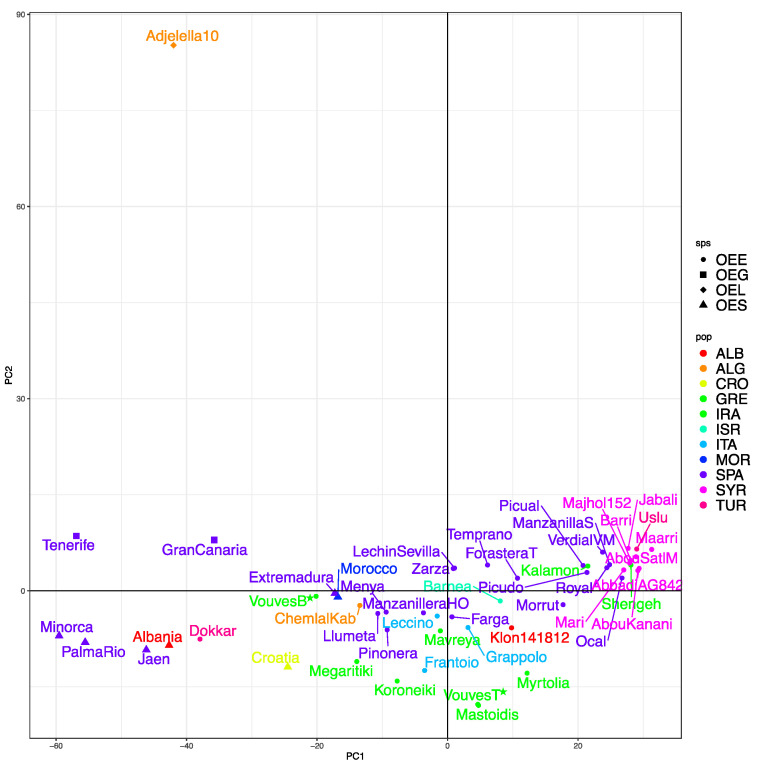
Principal Component Analysis (PCA) of the distance matrix of the different varieties used in this study. PC1 represents 18.77% of the variance and PC2 5.46%. Subspecies is encoded with the shape of the point (OEL, a diamond, for *Olea europaea* subsp. *laperrinei*; OEG, a square, *Olea europaea* subsp. *guanchica*; OES, a triangle, *Olea europaea* var. *sylvestris* and OEE, a circle, *Olea europaea* subsp. *europaea*) while country of origin is encoded with different colors (ALB (red), Albania; ALG (orange), Algeria; CRO (yellow), Croatia; GRE (light green), Greece; IRA (green), Iran; ISR (green-blue), Israel; ITA (light blue), Italy; MOR (blue), Morocco; SPA (purple), Spain; SYR (light purple), Syria and TUR (pink), Turkey). Samples of interest (Vouves bottom and top) have been marked with a star.

A PCA analysis on the samples show similar results (Figure 3). No cultivated subspecies or wild varieties such as *O. europaea* subsp. *laperrinei* (‘Adjelella10’), *O. europaea* subsp. *guanchica* (‘Tenerife’ and ‘Gran Canaria’) and *O. europaea* var. *sylvestris* (‘Minorca’, ‘PalmaRio’, ‘Jaen’, ‘Albania’, ‘Croatia’, ‘Extremadura’ and ‘Morocco’) appear to be separated from the main cluster of cultivated olives (*O. europaea* subsp. *europaea*). ‘Dokkar’ is close to the oleaster accessions, indicating a possible gene flow with the wild populations. The bottom of the Vouves tree is also close to the oleaster accessions, while the sample from the top of the tree clusters with the Greek accession ‘Mastoidis’ (Figure 3).

The topology of the phylogenomic tree is similar to previously published phylogenomic trees [31,33], with Italian and Syrian/Iranian samples as monophyletic groups, Spanish samples grouped in two branches with one of them being a sister group to the Syrian/Iranian groups. Greek samples were also distributed in a similar fashion with ‘Kalamon’ grouped with the Syrian samples and ‘Myrtolia’, ‘Mastoidis’ and ‘Koroneiki’ comprising a monophyletic group sister to the Italian one. ‘Megaritiki’ appeared as one of the most outer taxa of the cultivated olives similarly to ‘Dokkar’, so it is possible that this cv has some contribution from the wild olive populations [31,33].

**Figure 4:**
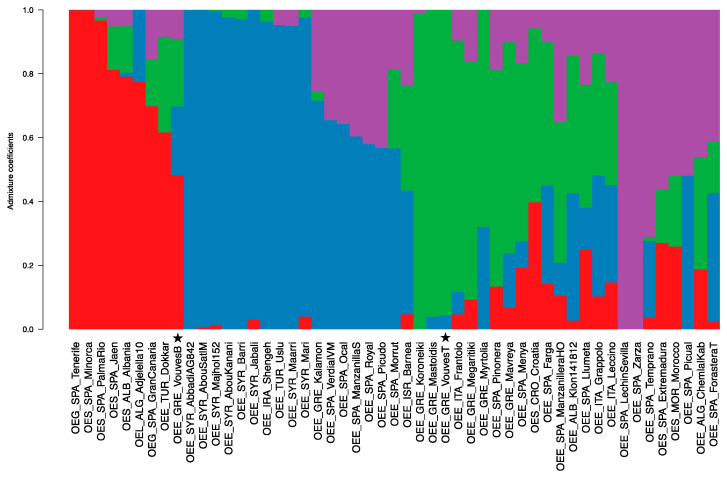
Admixture analysis for K = 4. Four populations are represented with the following colors: Red (noted in the text as G1) composed mostly by wild accessions (*Olea europaea* subsp. *laperrinei*; *Olea europaea* subsp. *guanchica*; and *Olea europaea* var. *sylvestris*); Purple (noted in the text as G2) has southern Spain accessions and some *Olea europaea* var. *sylvestris*; Blue (noted in the main text as G3) is composed by individuals from Syria and Iran; Green (noted in the text as G4) present-day Greek and Italian accessions and some accessions from north-western Spain. Vouves tree samples have been marked with a star.

The clustering analysis using STRUCTURE software and DAPC evidenced four clusters as the most probable number (Figure S2), with two, three and six being the alternative scenarios. The grouping of the different samples using four groups with Structure showed a first group (G1) composed by non-cultivated olives such as *O. europaea* subsp. *laperrinei*, *O. europaea* subsp. *guanchica* and most of the *O. europaea* var. *sylvestris* with the exception of the ‘Extremadura’, ‘Morocco’ and the ‘Croatia’ accessions that cluster in the groups “G2”, “G2” and “G4”, respectively. ‘Dokkar’ and the sample of the bottom of the Vouves tree also cluster in the group “G1”. Both samples show some component of the group “G4”. The second group, “G2”, is composed by accessions from southern Spain such as ‘Lechin de Sevilla’, ‘Zarza’ and ‘Temprano’. In this group, there can also be found accessions with some components of the groups “G1 + G4” such as ‘Chemlal Kabile’ and “G4 + G3” such as ‘Forastera T.’. A third group, “G3”, is composed by most of the cvs from Syria such as ‘Abou Kanami’, ‘Mari’ and ‘Barri’. In this group, there can also be found the Greek sample ‘Kalamon’ as well as some southern Spain samples such as ‘Verdial’, ‘Ocal’ or ‘Picudo’ as admixture between this “G3” group and the “G2”. The fourth group, “G4” is composed mostly by Greek, Italian and Northwest Spanish accessions including the top of Vouves tree sample (Figure 4). The results for the DAPC analysis are similar (Figure S3). The clustering analysis is also similar to the previously published analysis if the groups G2 and G3 are considered as a single group [33] or G1 and G2 are considered as one group and then G3 and G4 as another one [31]. A recent work with a wider sampling of wild accessions evidenced two separate groups; one comprising *O. europaea* var. *sylvestris* and another comprising *O. europaea* subsp. *guanchica* [40] in contrast to the present work where only one group is detected. This could evidence one of the limitations of the sampling of the present study associated with non-representative number of accessions for some groups, such as *guanchica*. Nevertheless, the *sylvestris* accessions appears as a group separate from the group of cvs giving enough resolution to distinguish both groups even if this study did not sample hundreds of accessions. 

Examining the composition of the individuals in admixture analysis it appears possible that some introgression and gene flow has taken place between the wild and some cultivated accessions such as “Dokkar”. Consequently, and in order to test this possibility, an ABBA-BABA analysis was performed using the Italian varieties as sister group, the wild accessions “Minorca”, “Palma del Rio” and “Jaen. The *Olea europaea* subsp. *laperrinei* was used as outgroup. The results evidenced that three cvs (“Chemlal Kabil”, “Megaritiki” and “Dokkar”) experienced introgression from wild olive trees: (Table S1). This is in agreement with the position of these three cvs in the phylogenetic tree (Figure 2a). The Vouves tree bottom sample did not present any *Olea europaea* var. *sylvestris* introgression according to the ABBA-BABA analysis.

The relation between the top of the Vouves tree sample and the Greek accession ‘Mastoidis’ was tested calculating the relatedness Ajk statistic between all the samples (Figure S4). Individuals with themselves will have values of 1 or higher, individuals in the same population will have values close to 0 and unrelated individuals will have negative values. The top of the Vouves tree sample has an Ajk statistics value of 0.53 with the ‘Mastoidis’ accession indicating a relation of the same order than half-siblings, and verifying that the Vouves tree was grafted with a present-day cv. This is in full agreement with present SSR analysis whereupon Vouves top sample fell within the ‘Mastoidis’ cluster (Figure 2b). The Vouves rootstock (bottom sample) showed a clear grouping with the oleaster samples in a similar way that the ‘Megaritiki’ Greek cv. A previous work hypothesized that ‘Megaritiki’ has an introgression of oleaster populations [31]. This result agrees with the Ajk statistics with values of 0.13, 0.10, 0.10, 0.09 and 0.09 with the ‘Croatia’, ‘Palma del Rio’, ‘Minorca’, ‘Jaen’ and ‘Albania’ oleaster accessions, respectively. The Vouves rootstock (bottom sample) shows higher values with the oleaster samples, especially with ‘Minorca’ (0.16), ‘Albania’ (0.15) and ‘Palma del Rio’ (0.14). These values are similar to the values that the oleaster samples show between them. Even though this agrees with the hypothesis that this sample could have oleaster introgressions, the estimated age of the tree could indicate an early stage of domestication and diversification from the oleaster accessions. A more extensive sampling of other Greek accessions as well as other oleaster samples of the West Mediterranean area could help to clarify this result. Greek cvs whose genomes were available for inclusion in the present study (Table 1; ‘Kalamon’, ‘Koroneiki’, ‘Mastoidis’, ‘Mavreya’, ‘Megaritiki’, ‘Myrtolia’) didn’t show any high Ajk values with the Vouves bottom sample so it cannot be documented that the original monumental tree has a special role in the development of these present-day Greek varieties. Nevertheless, previous SSR studies showed that the Vouves tree is genetically related with other “monumental” trees from the ‘Sotira’ area in Cyprus [24] in addition to an “ancient” tree from Peloponnese)—a region in continental Greece (present study). Both localities are geographically isolated from Crete, by sea, the first by appr. 700 Km and the second by appr. 300 Km. Similar to the Vouves bottom sample, the Sotira area “monumental” samples appear genetically remote by comparison to present-day local, Cypriot cvs. Specifically, [25] had performed Maximum Likelihood (ML) clustering analysis, employing 17 SSR loci data, of 51 old rootstocks (also dubbed ‘living fossils’ or ‘centennial olive germplasm’) and 12 present-day cvs from Cyprus. They showed that the ‘Vouves tree’ (‘Vouves bottom’ of present study) is genetically related to other monumental trees of the Sotira area in Cyprus (the two islands of Cyprus and Crete are 700 Km apart). The same authors subsequently performed coalescent modelling employing the same data as for ML. Similar to what is found for ‘Vouves bottom’ in the present study, [25] concluded that “most of the rootstocks were positioned externally to the core of the olive entries, thus underlining their lack of genetic affinity, but without ruling out the possible contribution to the establishment of the current cultivars”.

Overall, we believe that we have shown that the bottom of the Vouves tree is a well-supported separate branch with no gene flow from the *sylvestries* trees indicating that probably its cultivation was a separate event and that, at another level, wild cultivars from the eastern cluster together with those from the western Mediterranean basin.

The analysis of the Vouves tree can bring forth some interesting points about the date of the early diversification of the East/West cultivated populations. The ‘Farga’ taxon represents an ancient branch of domesticated olive trees dated between 300 and 1000 years old [41]. The phylogenetic tree shows a clear divergency of some lineages (e.g., Italian or some Greek popular accessions) from the ‘Farga’ lineage indicating that the ancestor of these cvs could have existed more than a thousand years ago. The estimated age of diversification between Eastern and Western cultivated populations is dated around 6000 years ago [5]. The position of the bottom of the Vouves tree sample, dated more than 4000 years ago could indicate a later diversification (appears as an outgroup for the cultivated olives without any apparent gene flow with the oleaster populations) although more ancient monumental trees should be studied before any solid conclusion is drown.

Taking together the conclusions of [25,41] and of the present study it could be proposed that, in the Mediterranean Basin, there existed an ancient olive tree common genetic pool which is only partly represented in few present-day cvs.

### 2.3. Gene Space Variation in the Vouves Monumental Olive Tree

The genome resequencing allows for analysis of possible changes occurred in the gene space of a genome. Most of the variants between the reference genome (Oe451) and the Vouves tree samples were intergenic variants (55.30% and 46.53% of the annotated variants for bottom and top samples, respectively), followed by 5 Kb downstream (14.90% and 12.98%) and upstream gene variants (20.63% and 16.81%). Intron variants accounted for 5.12% and 4.15% of the total variants in both datasets. The impact of the variants in the Vouves samples was compared with other Greek genotypes (Table 2). Between 36.73% (Vouves Top) and 56.36% (‘Megaritiki’) of the genes presented at least one variant with high impact. Most of these types of variants are associated with frameshift or gaining of a stop codon (Table 3).

**Figure 5:**
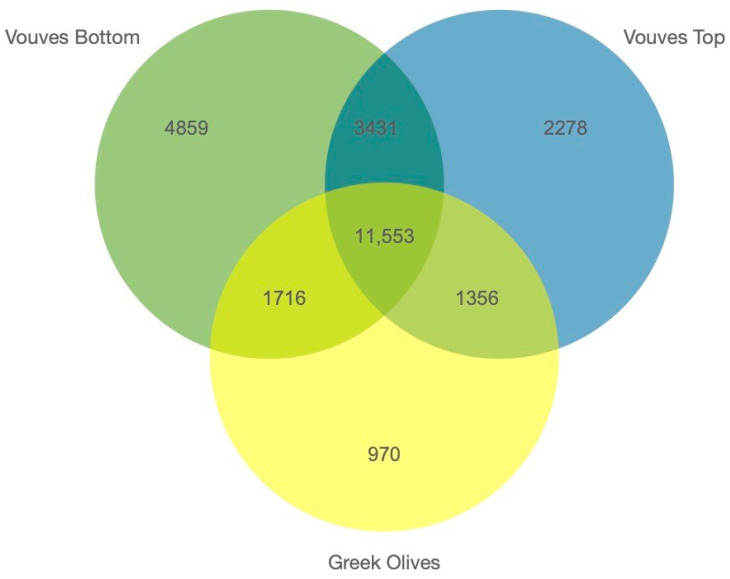
Venn diagram showing the number of High Impact (HI) variants shared between the Vouves tree bottom (green), the Vouves tree top sample (blue) and the other Greek accessions (yellow).

**Figure 6:**
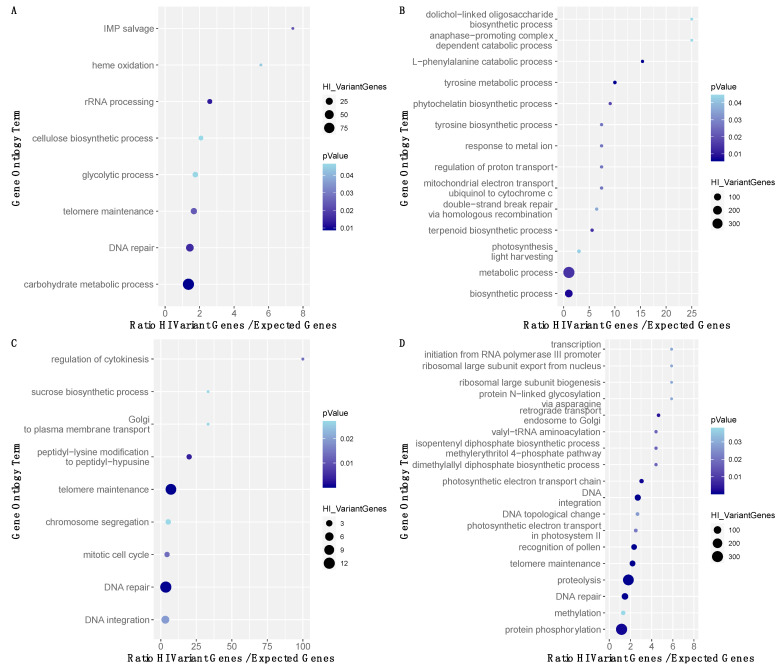
Gene Ontology analysis of the genes affected by high impact variants. The x-axis represents the value of the over or under-representation of these categories. The size of the dot represents the number of genes of the category and the color intensity its statistical significance as *p*-value. (**A**) Vouves bottom tree sample; (**B**) Vouves top tree sample; (**C**) High impact changes shared by all the Greek accessions (‘Kalamon’, ‘Koroneiki’, ‘Mastoidis’, ‘Mavreya’, ‘Megaritiki’ and ‘Myrtolia’) but absent in the Vouves tree; (**D**) Genes affected by high impact variants common for all the datasets (Vouves tree and Greek accessions).

In the present study, 11,553 genes with high impact variants were shared between all the Greek accessions and the Vouves tree samples. Indeed, 13,269 and 12,909 genes with high impact variants were shared between the Greek accessions and the bottom and the top Vouves tree sample, respectively, meanwhile 14,984 genes were shared between both Vouves samples (Figure 5). To further understand the processes in which these genes may be involved, a Gene Set Enrichment Analysis (GSEA) was performed in the different groups. The first group, formed by 4859 genes with high impact variants in the Vouves bottom tree sample, presented an enrichment in eight terms: “IMP Salvage” (GO: 0032264), “Heme Oxidation” (GO: 0006788), “rRNA processing” (GO: 0006364), “cellulose biosynthetic process” (GO: 0030244), “Glycolytic process” (GO: 0006096), “Telomere maintenance” (GO: 0000723), “DNA repair” (GO: 0006281) and “Carbohydrate metabolic process” (GO: 0005975) (Figure 6A). The second group had 2,278 genes with high impact variants for the Vouves top tree sample. It presented enrichment in the following terms “Regulation of proton transport” (GO: 0010155), “Mitochondrial electron transport ubiquinol to cytochrome c” (GO: 0006122), “Terpenoid biosynthetic process” (GO: 0016114), “Response to metal ion” (GO: 0010038), “Biosynthetic process” (GO: 0009058), “L-phenylalanine catabolic process” (GO: 0006559), “tyrosine metabolic process” (GO: 0006570), “tyrosine biosynthetic process” (GO: 0006571), “Metabolic process” (GO: 0008152), “Double-strand break repair via homologous recombination” (GO: 0000724), “Anaphase-promoting complex dependent catabolic process” (GO: 0031145), “Dolichol-linked oligosaccharide biosynthetic process” (GO: 0006488), “Phytochelatin biosynthetic process” (GO:0046938) and “Photosynthesis light harvesting” (GO: 0009765) (Figure 6B).

The third group had 970 genes associated with high impact changes shared by all the Greek accessions (‘Kalamon’, ‘Koroneiki’, ‘Mastoidis’, ‘Mavreya’, ‘Megaritiki’ and ‘Myrtolia’) but absent in the Vouves tree. This cluster presented an enrichment in the following Gene Ontology Terms: “Mitotic cell cycle” (GO: 0000278), “DNA repair” (GO: 0006281), “Golgi to plasma membrane transport” (GO: 0006893), “ATP metabolic process” (GO: 0046034), “Chromosome segregation” (GO: 0007059), “Sucrose biosynthetic process” (GO: 0005986), “DNA integration” (GO: 0015074), “Regulation of cytokinesis” (GO: 0032465), “Peptidyl-lysine modification to peptidyl-hypusine” (GO: 0008612) and “Telomere maintenance” (GO: 0000723) (Figure 6C). The list of the genes as well as the variants and their impacts have been summarized in the Table S2. The fourth group contained 11,553 genes with high impact variants common for all the datasets (Vouves tree and Greek accessions). This group presented a GO enrichment for the terms “valyl-tRNA aminoacylation” (GO: 0006438), “ribosomal large subunit biogenesis” (GO: 0042273), “ribosomal large subunit export from nucleus” (GO: 0000055), “dimethylallyl diphosphate biosynthetic process” (GO: 0050992), “DNA integration” (GO: 0015074), “proteolysis” (GO: 0006508), “DNA topological change” (GO: 0006265), “DNA repair” (GO: 0006281), “photosynthetic electron transport chain” (GO: 0009767), “photosynthetic electron transport in photosystem II” (GO: 0009772), “methylation” (GO: 0032259), “transcription DNA-templated” (GO: 0006351), “isopentenyl diphosphate biosynthetic process methylerythritol 4-phosphate pathway” (GO: 0019288), “transcription initiation from RNA polymerase III promoter” (GO: 0006384), “recognition of pollen” (GO: 0048544), “retrograde transport endosome to Golgi” (GO: 0042147), “protein *N*-linked glycosylation via asparagine” (GO: 0018279), “protein phosphorylation” (GO: 0006468) and “telomere maintenance” (GO: 0000723) (Figure 6D).

The comparison of the Gene Ontology Terms of the genes with high impact variants between the Greek samples and the Vouves bottom and top tree didn’t show any term related with fatty acid metabolism or accumulation. This comparison didn’t reveal other terms related with adaptation to biotic or abiotic stresses. Nevertheless, it is interesting that pathways related with carbohydrate and amino acid metabolism were found, in the same way that has been found in the sweep regions related with the process of domestication [31]. An important proportion of these genes are related with the cell wall biosynthesis (e.g., “cellulose synthase 1” or “heteroglycan glucosidase 1”). The impact of these variations in the phenotype of the different varieties is uncertain. These genes are part of extensive gene families (e.g., 42 genes were annotated as “cellulose synthase” in the reference genome used) while mutations in some of the copies can be a part of the process of non-functionalization of multiple gene copies that may be risen by duplications. The study of the evolution of the cellulose synthase revealed that some of them are indeed pseudogenes [42] so it is expected to find high impact variants on those. It is also interesting to find a high number of genes with HI variants associated with DNA repair. Most of these genes have the descriptor “PIF1 helicase”. PIF1 DNA helicases are important players in the stability of the genome [43], but their multiple copies and their redundancy has limited the number of functional studies associated to this gene family. The olive genome has annotated 103 genes under the descriptor of “PIF1 helicase”. Indeed, 9, 2, 7 PIF1 Helicase genes presented high impact variations in the Vouves bottom and top samples and the other Greek accessions, respectively. One possibility of this elevated number of PIF1 genes as well as the high impact variations on those may be related with the fact that Helitron HelRel proteins have an S1 helicase domain similar to the PIF1 helicase [44]. This may lead to the misidentification of these helitrons as PIF1 helicases.

## 3. Conclusions

The olive tree of Vouves, which is considered to be one of the oldest olive trees in the world, was studied to reveal its genetic relations with present-day cultivated and wild genotypes and to shed light into potential origin or routes of domestication of olive. The top of the Vouves tree sample has an Ajk statistics value of 0.53 with the ‘Mastoidis’ accession indicating a relation of the same order than half-siblings, and verifying that the Vouves tree was grafted with a present-day cv. The Vouves rootstock (bottom sample) showed a clear grouping with the oleaster samples in a similar way that the ‘Megaritiki’ Greek cv. An ABBA-BABA analysis showed that it doesn’t have any introgression from the oleaster population. The Greek cvs didn’t show any high Ajk values with the Vouves bottom sample so it cannot be documented that the original monumental tree has a special role in the development of some important present-day Greek varieties. Nevertheless, previous results showed that the Vouves tree is genetically related with other monumental trees of the Sotira area in Cyprus. An ongoing survey on centennial olive trees allover Greece and the comparative analysis of resequencing data with other projects will shed more light on the history of olive growing and potentially reveal ancient varieties to be reserved as natural heritage and genotypes with desirable traits to be exploited as invaluable pre-breeding material.

## 4. Materials and Methods

### 4.1. Plant Samples and DNA Extraction

Fresh healthy leaves were collected during July 2018 separately (i) from the highest part of the Vouves monumental olive tree (referred as ‘top’) and (ii) from the lowest part of the tree at the base of the trunk (referred as ‘bottom’) and were immediately transferred in a cool container to the laboratory for DNA extraction. For all analysis described herein, total genomic DNA was isolated from leaf material using the DNeasy Plant Mini kit (Qiagen cat. No 69104, Düsseldorf, Germany) according to manufacturer’s instructions. The hypothesis that the tree is consisted of two genotypes united through grafting was tested.

### 4.2. DNA Sequencing and SSR Analysis

1 ug of DNA for each of the Vouves monumental olive tree (top and bottom) were sent to Novogene Co. Ltd. (Sacramento, CA, USA, https://en.novogene.com/, accessed on 25 June 2018) for whole genome sequencing. The libraries were prepared following the standard Illumina Nextera^®^ protocol for insert sizes ~350 bp. Then, they were sequenced with an Illumina Novaseq6000 platform, paired-end 150 bp.

SSR analysis followed [45] with the inclusion, in the SSR set, of two new loci while omitting one, previously used. Total SSR loci presently employed were 11 (DCA3, DCA5, DCA9, DCA14, DCA16, DCA18, EMO90, GAPU71B, GAPU101, GAPU103A, UDO99). Allelic data were used to construct a UPGMA similarity dendrogram based on Jaccard distances, similarly to [36].

### 4.3. Read Processing, Reference Mapping and Variant Calling

Reads were processed with Fastq-mcf v1.04.676 from the Ea-utils package [46], then they were mapped with BWA v0.7.17-r1188 [47] to the *Olea europaea* var. *sylvestris* genome reference v1.0 [32] downloaded from Phytozome (https://phytozome.jgi.doe.gov/, accessed on 20 May 2019) [48]. Mapping coverage was evaluated with BEDtools genomecov v2.29.0 [49] with the option -bga. Variants were called using Freebayes v1.3.1-16-g85d7bfc [50] with a minimum read coverage of 5, a minimum mapping quality of 20 and discarding complex variants. Non-biallelic variants were filtered with Vcftools v0.1.15 [51]. The impact of the variants was evaluated with Snpeff v4.3 [52].

### 4.4. Origin Analysis

RNA-Seq reads representing 55 olive accessions from 14 different countries were downloaded from GenBank SRA (ProjectID PRJNA525000 [6] as well as 50 olive accessions Whole Genome DNA Resequencing (WGR) data from the SRA project PRJNA556567 [33]. Reads were processed with Fastq-mcf v1.04.676 from the Ea-utils package [46] and them mapped to the reference genome *Olea europaea* var. *sylvestris* genome reference v1.0 [32] using Hisat2 v2.1.0 [53]. Variants were called using Freebayes v1.3.1-16-g85d7bfc [50] with a minimum read coverage of 5, a minimum mapping quality of 20 and discarding complex variants. The VCF file was filtered using Vcftools v0.1.15 [51] removing the variants that were not present in all the samples and keeping only biallelic Single Nucleotide Polymorphisms (SNPs). The VCF file was upload in RStudio v1.1.463 running R v3.5.1 using Adegenet v2.1.1 [54] and Poppr v2.8.3 [55] packages. A distance matrix between all the samples’ SNP was calculated with the function dist() with the default parameters. A distance tree was calculated with the function aboot() function from the Poppr package using Nei distance, NJ tree and 1000 samples. A Principal Components Analysis (PCA) was performed using the prcomp() function from the Stats R core package with the default parameters and it was plotted with the Ggplot2 v3.2.0 package. The DAPC analysis was performed with the function dapc() from the Adegenet package.

Population structure was inferred using two alternative procedures: (1) a Bayesian, model-based algorithm employed through STRUCTURE software (release: V2.3.4, July 2012) [56] and (2) Discriminant Analysis of Principal Components [57] which produces genetic clusters using a few “synthetic” variables constructed as linear combinations of the original variables (alleles). These alleles are in turn selected as having the largest between-group variance and the smallest within-group variance.

The ABBA-BABA analysis was performed using the same VCF file that was described before. The VCF files was converted to the EIGENSTRAT format with the script convertVCFtoEigenstrat.sh from Joanam at Github (https://github.com/joanam/scripts/blob/master/convertVCFtoEigenstrat.sh/, accessed on 8 September 2021). The “admixr” R package v0.9.1 was used to perform the ABBA-BABA analysis. In summary, the EIGENSTRAT files were uploaded in R with the eigenstrat() function. The sister group to the targets was the Italian cvs (“Frantoio”, “Grappolo”, “Leccino) being a monophyletic branch in the Figure 2. The wild accessions were the *O. europaea* var. *sylvestris* accessions: “Minorca”, “Jaen”, “PalmaRio” being also a monophyletic clade for *sylvestris*. Finally, as outgroup the *O. europaea* subsp. *laperrinei* was used.

### 4.5. Gene Space Comparison

Gene space comparison was performed using three different approaches by comparing the two Vouves olive samples, the cv ‘Farga’ (SRA accession ERR1346608) [30] and the wild type reference *Olea europaea* var. *sylvestris* genome v1.0 [32]. For the first approach, the presence or absence of the genes annotated in the reference genome was analyzed using BEDtools v2.29.0 [49]. First the BAM files containing the mapped reads was converted to BED with the function bamtobed. Then, the bed rows were merged using the function merge. Finally, the function complement was applied to the previous BED file and the reference gene annotation GFF file, producing a filtered GFF with the genes that were not covered by the mapped reads. For the second approach the impact of the variants (see Section 3) on the gene space was evaluated with Snpeff v4.3 [52].

## Figures and Tables

**Table 1:** Different type of variants determined following comparison of the two Vouves samples and six previously sequenced Greek cvs with the genome of *Olea europaea* var. *sylvestris* version Oe451.

Sample	Total Variants (M)	Heterozygous Variants/100 bp	Total Variants/100 bp	SNP (M)	InDels (M)	MNP (M)
Vouves bottom ^1^	23.26	1.31	2.09	18.79	1.27	0.12
Vouves top ^1^	19.54	1.42	1.76	15.73	1.09	0.09
Kalamon ^2^	29.47	1.80	2.62	19.26	1.09	0.12
Koroneiki ^2^	34.38	2.07	3.07	22.38	1.26	0.19
Mastoidis ^2^	29.70	1.76	2.65	19.40	1.09	0.17
Mavreya ^2^	31.80	1.99	2.84	21.32	1.18	0.12
Megaritiki ^2^	35.70	2.16	3.20	23.24	1.30	0.18
Myrtolia ^2^	29.10	1.69	2.60	18.75	1.08	0.16

Note: ^1^—Samples resequenced in this publication; ^2^—Samples downloaded from the NCBI SRA public repository from the cite 33.

**Table 2:** Percentage of the different variant categories produced in the variant annotation for each of the samples.

Sample	% Intergenic Variants	% 5 Kb Upstream Variants	% 5 Kb Downstream Variants	%Intron Variants	% Genic Variants			Genes with HI Variants
					High Impact ^1^	Moderate Impact ^2^	Low Impact ^3^	
Vouves bottom	55.30	20.63	14.90	5.12	0.23	2.26	1.56	21,559
Vouves top	46.53	16.81	12.98	4.15	0.19	1.87	1.31	18,618
Kalamon	59.45	18.32	13.72	4.46	0.27	2.32	1.45	25,917
Koroneiki	59.93	18.25	13.64	4.32	0.26	2.20	1.40	26,334
Mastoidis	60.12	18.01	13.56	4.34	0.26	2.27	1.43	25,041
Mavreya	59.77	18.11	13.69	4.43	0.27	2.28	1.44	26,558
Megaritiki	59.45	18.43	13.76	4.45	0.26	2.23	1.40	28,567
Myrtolia	59.79	18.28	13.67	4.32	0.26	2.26	1.42	26,164

Notes: ^1^—High impact (HI) variants are defined as those variants with exon lost, frameshift, splice acceptor or donor variant, loss of the start or gain of a stop of the translation. ^2^—Moderate impact (MI) variants are those with a conservative or disruptive in-frame insertions or deletions, missense variants and splice region variants. ^3^—Low impact (LI) variants are those as codon initiation variants and synonymous variants and variants in the stop codon.

**Table 3:** Summary of the different sources of high impact variants affecting each of the Greek accessions.

Sample	Genes with High Impact Variants						
	Exon Lost	Frameshift	Splice Acceptor	Splice Donor	Start Lost	Stop Gain	Stop Lost
Vouves Bottom	2	12,651	4697	4632	2112	11,624	3126
Vouves Top	1	10,588	3781	3723	1792	9748	2598
Kalamon	1	14,752	5954	5790	2860	17,086	3603
Koroneiki	2	16,355	6503	6204	3275	17,037	4003
Mastoidis	3	14,896	5798	5570	2757	16,170	3593
Mavreya	5	15,829	6318	6071	3111	17,307	3736
Megaritiki	3	16,924	6956	6629	3386	19,322	4199
Myrtolia	1	14,565	5789	5451	2747	16,332	3427

## Data Availability

The two Vouves trees re-sequencing data was submitted to the NCBI SRA database with the BioProject accession number PRJNA721943.

## Supplementary Materials

The following are available online at https://www.mdpi.com/article/10.3390/plants10112374/s1, Figure S1: PCA analysis of the genetic distances between the different samples. Samples with SNPs produced from RNASeq data have the prefix “t”. Samples with SNPs produced by DNA WGR data have the prefix “g”. Figure S2: Most probable number of clusters based in the lowest entropy value for the DAPC analysis. Number of ancestral populations are compared with the entropy values; Figure S3: Assignment of the different individuals to the ancestral populations for K = 2, K = 3, K = 4 and K = 5 for the DAPC analysis; Figure S4: Analysis of the relatedness between the different samples based in the AJK index. Positive relationships are colored in red and negative relationship are in blue; Table S1: ABBA-BABA analysis performed to compare individuals with possible admixture with the groups Italian cvs (X), *O. europaea* var. *sylvestris* (Y) and *Olea europaea* subsp. *perrinei* accession used as outgroup (Z). Significant values for the introgressions from *O. europaea* var. *sylvestris* have been colored in green. Significant values for the introgressions from the Italian cvs have been colored in blue including the name of the accession; Table S2: List of genes associated with the GSEA for genes containing High Impact (HI) variants.

## Abbreviations

AJK	Averaged pairwise relatedness index
BED	Browser Extensible Data
DAPC	Discriminant Analysis of Principal Components
GBS	Genotyping-By-Sequencing
GFF	Generic Feature Format
GO	Gene Ontology
GSEA	Gene Set Enrichment Analysis
HI	High Impact variant
LI	Low Impact variant
MI	Moderate Impact variant
PCA	Principal Component Analysis
VCF	Variant Call Format
cv(s)	cultivar(s)
