# Supplementary figures and images for: Elucidation of the Origin of the Monumental Olive Tree of Vouves in Crete, Greece
Source: Plants (Basel). 2021 Nov 4;10(11):2374. doi: 10.3390/plants10112374 (PMC8620074; doi:10.3390/plants10112374)

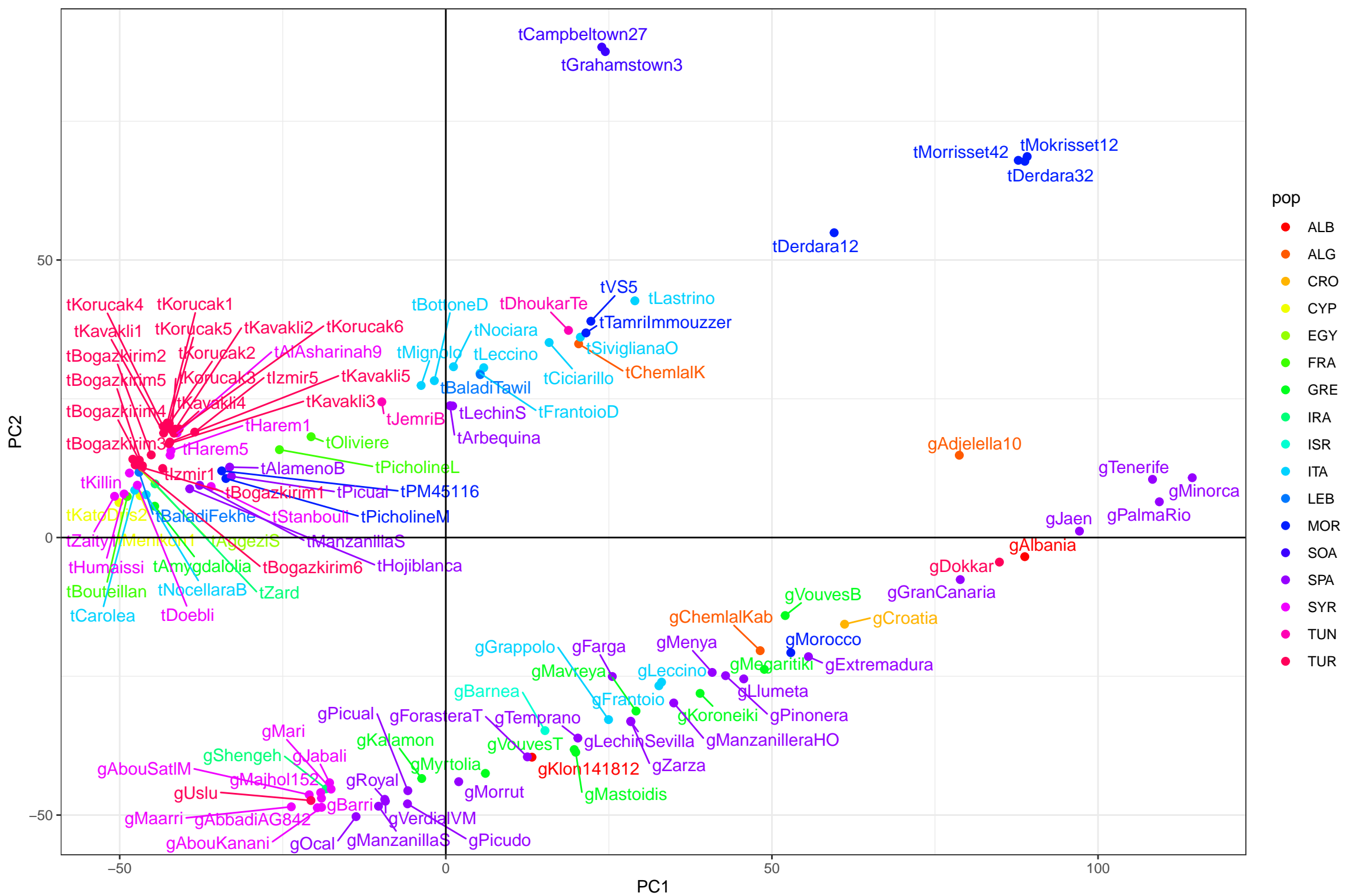

Supplement: Supplementary file 1 [file plants-10-02374-s001.zip › Figure S1.pdf]

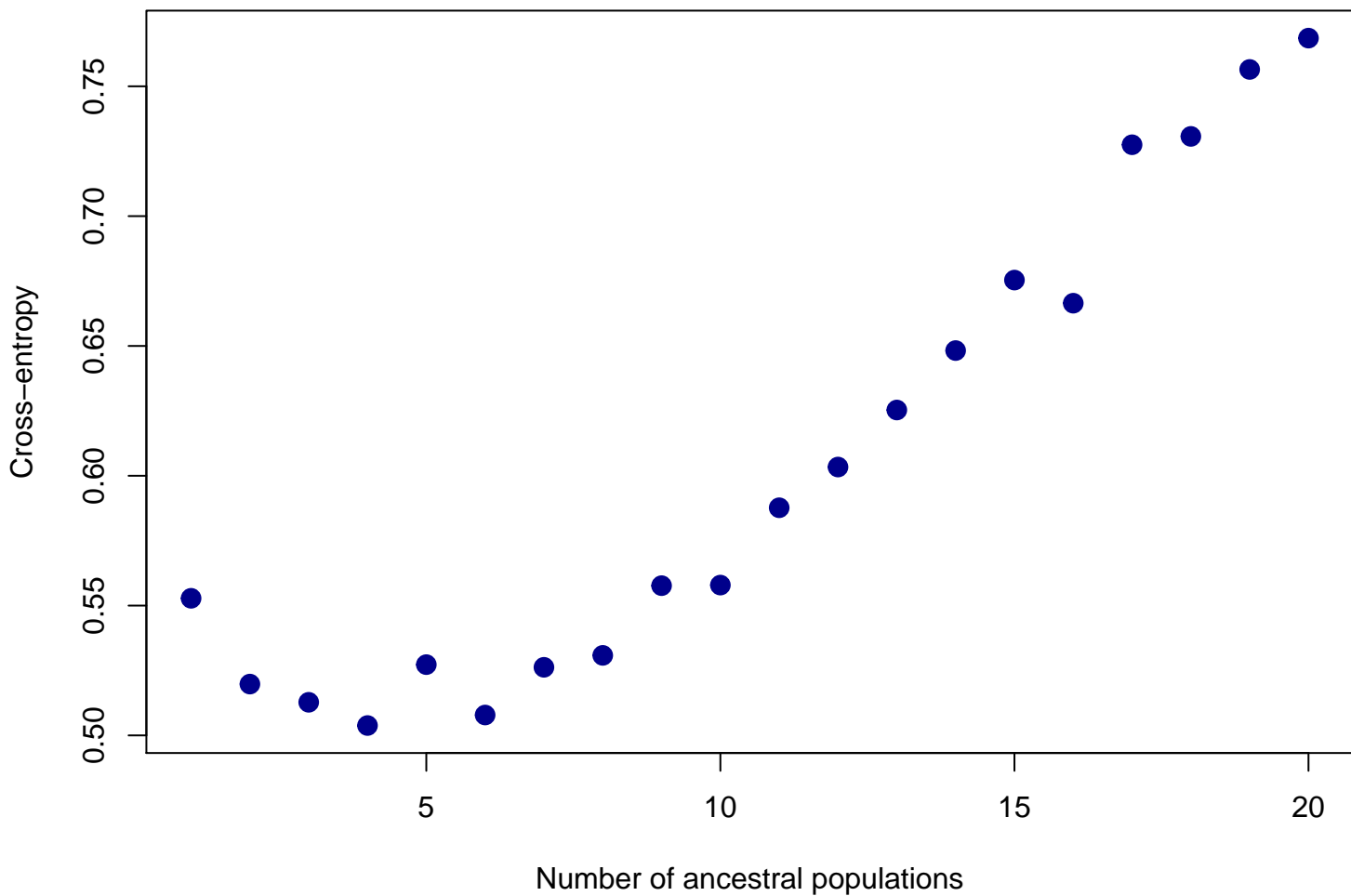

Supplement: Supplementary file 1 [file plants-10-02374-s001.zip › Figure S2.pdf]

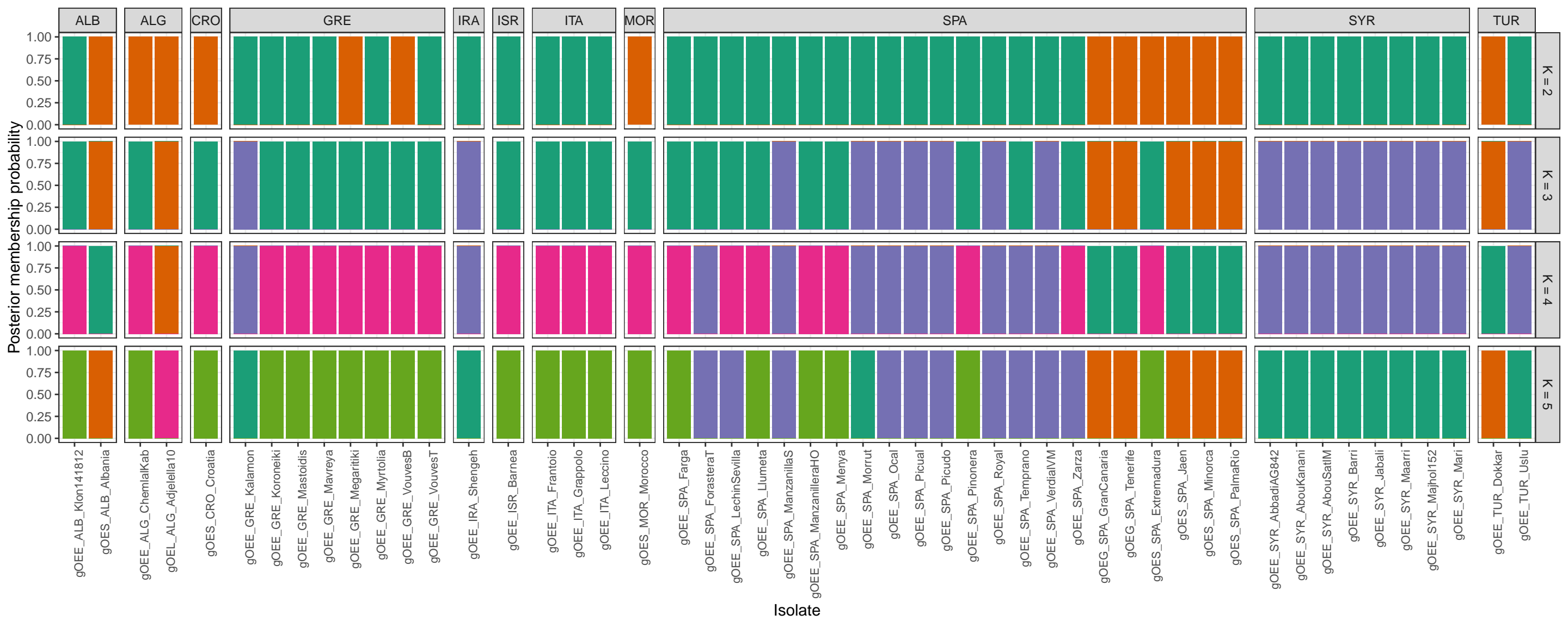

Supplement: Supplementary file 1 [file plants-10-02374-s001.zip › Figure S3.pdf]

INDV2

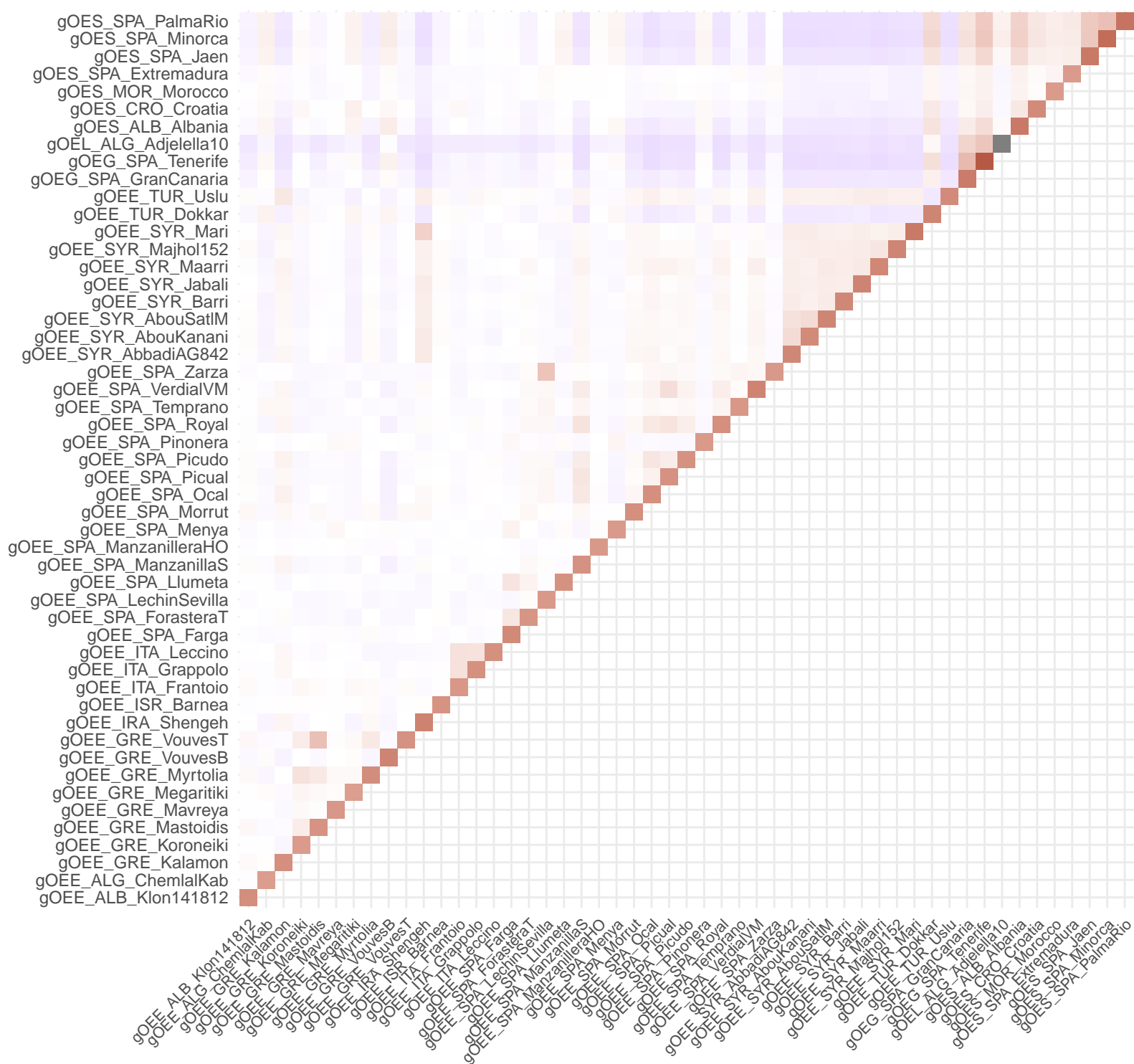Relatedness  
AJK index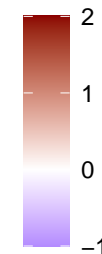

INDV1

Supplement: Supplementary file 1 [file plants-10-02374-s001.zip › Figure S4.pdf]
